# Potentiating Antigen-Specific Antibody Production with Peptides Obtained from In Silico Screening for High-Affinity against MHC-II

**DOI:** 10.3390/molecules24162949

**Published:** 2019-08-14

**Authors:** Yoshiro Hanyu, Yuto Komeiji, Mieko Kato

**Affiliations:** 1 Structural Physiology Research Group, Biomedical Research Institute, National Institute of Advanced Industrial Science and Technology (AIST), 1-1-1 Higashi, Tsukuba 305-8566, Japan; 2 Department of Biochemistry, Bio-Peak Co., Ltd., 584-70 Shimonojo, Takasaki 370-0854, Japan

**Keywords:** monoclonal antibody, MHC-II, agretope, in vitro immunization, in silico screening, IgG

## Abstract

Monoclonal antibodies with high affinity and specificity are essential for research and clinical purposes, yet remain difficult to produce. Agretope peptides that can potentiate antigen-specific antibody production have been reported recently. Here, we screened in silico for peptides with higher affinity against the agretope binding pocket in the MHC-II. The screening was based on the 3D crystal structure of a complex between MHC-II and a 14-mer peptide consisting of ovalbumin residues 323–339. Using this 14-mer peptide as template, we constructed a library of candidate peptides and screened for those that bound tightly to MHC-II. Peptide sequences that exhibited a higher binding affinity than the original ovalbumin peptide were identified. The peptide with the highest binding affinity was synthesized and its ability to boost antigen-specific antibody production in vivo and in vitro was assessed. In both cases, antigen-specific IgG antibody production was potentiated. Monoclonal antibodies were established by in vitro immunization using this peptide as immunostimulant, confirming the usefulness of such screened peptides for monoclonal antibody production.

## 1. Introduction

Monoclonal antibodies are indispensable for research, diagnostics, and therapeutics. For effective use in these fields, antibodies that exhibit high affinity and specificity toward antigens are required. However, establishing an antibody with the desired affinity and specificity against different types of antigens remains a challenge. In particular, it remains difficult to obtain useful antibodies against poorly immunogenic antigens, such as those that show high homology to proteins of host animals, and antigens that are toxic to their hosts. To generate high-titer monoclonal antibodies against these poorly immunogenic antigens, strong immunopotentiators must be used to elicit an intense cellular immune response. Freund’s complete adjuvant (FCA), composed of dried and inactivated *Mycobacterium tuberculosis*, is a strong immune booster used widely in monoclonal antibody production [1]. Although FCA has been a mainstay in immunological research for decades, it exerts several undesirable side effects such as occasional inflammation and toxicity to the host animal. Therefore, immunostimulants that effectively activate the immune system, produce antibodies with high titer and selectivity, and cause weaker side effects than FCA are required [2]. Various immunostimulants and adjuvants have been used, including other microorganism-derived compounds such as cytokines, muramyl dipeptides (MDPs) and tripeptides, liposomes [3], aluminum compounds (e.g., alum), nanoparticles [4], and polymeric microspheres [5]; however, stimulation by these reagents was not adequately strong, and thus, an optimal stimulator remains to be identified. The immuno-stimulatory nucleic acid CpG oligodeoxynucleotide (CpG ODN) has also been used as an immunostimulant at the time of immunization [6,7,8]. CpG ODN is a short oligonucleotide that contains unmethylated cytosine-guanine dinucleotides that feature a specific base context. Exposure to CpG ODN results in extremely rapid and strong immune activation, and when applied together with an antigen, CpG ODN leads to production of high titers of antigen-specific antibodies.

Takatsu and Kariyone [9] determined that Peptide-25 derived from Ag85B of *Mycobacterium tuberculosis* induces Th1 development. Peptide-25 is a 15-mer peptide, (aa 240–254) of Ag85B (also known as αantigen and MPT59) [10]. It is a major T-cell epitope and is presented as an agretope by the major histocompatibility complex II (MHC-II) on the cell surface. This complex of Peptide-25 and MHC-II is recognized by the T cell receptor, which induces T cell activation. Peptide-25 is immunogenic in I-A^b^ mice and induces the development of Th1 cells that express TCRVβ11Vα5 [11]. Immunization of C57BL/6 mice (I-A^b^ mice) with an antigen together with Peptide-25 was shown to enhance antigen-specific IgG2a production. Thus, Peptide-25 exhibits potent adjuvant activity in both the humoral- and cell-mediated immune responses that appear to be mediated by Th1 cells [12]. We reported previously that antigen-specific antibody production was potentiated by Peptide-25, thus strongly stimulating the production of antigen-specific IgG1 [13]. This potentiation was remarkably high in BALB/c mice (I-A^d^ mice). We showed that T cells were activated by Peptide-25 through modulation of the Th1/Th2 balance during immunization. The observed potentiation suggests that externally applied Peptide-25 binds to the MHC-II and the ensuing complex activates helper T cells. As a result, helper T cells activate B cells in a polyclonal fashion and these activated B cells lead to increased antibody production. We synthesized several Peptide-25 mutants and studied their effects in immune signaling. Results showed that peptide’s affinity to the MHC-II molecules were crucial for potentiation effect.

Based on the above potentiation of antigen-specific antibody production by extracellular addition of an agretope peptide, we hypothesized that such antibody production could be more efficient if the agretope peptides bound more tightly to the MHC-II. To address this possibility, we performed an in silico screening to identify peptides with high affinity against the MHC-II. We then experimentally confirmed the potentiation effect of the selected peptides on antigen-specific antibody production.

## 2. Results

### 2.1. Screening the Agretope Peptides

**Figure 1:**
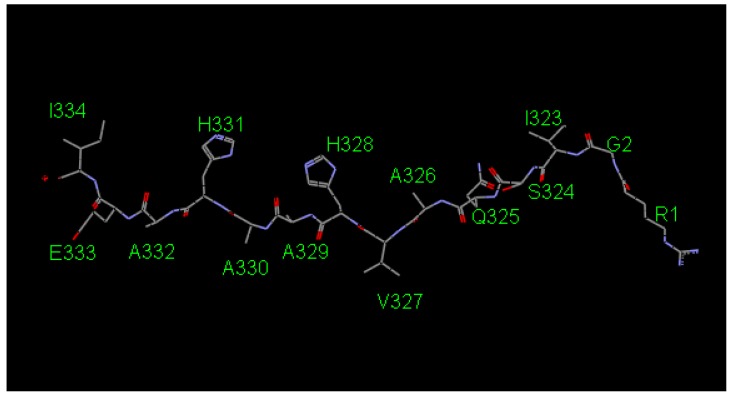
3D structure of the template OVApeptide excised from the crystal structure (1IAO).

To find peptides with high affinity against the MHC-II, in silico screening was performed. Using a 14-mer peptide consisting of amino acid residues 323–339 of ovalbumin (hereafter referred to as OVApeptide, Figure 1) as template, we tried to design peptides that bound to the MHC-II more tightly than the original OVApeptide.

AutoLudi (see Section 4.2) was performed based on the 3D crystal structure (PDB ID: 1IAO, [14]) of the complex of OVApeptide and I-A^d^, the MHC-II of BALB/c mice. Two-fold AutoLudi screening was performed as follows. In the first screening, all amino acid residues of the OVApeptide were replaced with Gly while preserving the 3D structure of the main chain. The resultant (Gly)_14_ peptide was used as the scaffold. Then, each Gly was automatically replaced with various amino acid residues, and the Ludi score was calculated for the resultant peptide while considering rotational conformations of the residues. Amino acid residues that showed a higher score than Gly were selected (Table 1).

Before the second screening, candidate amino acid residues were manually selected because it was practically impossible to investigate all the combinations of these candidate residues (5 × 6 × 8 × 5 × 4 × 4 × 3 × 8 × 1 × 1 × 8 × 2 × 5 × 2 = 73,728,000 combinations). Original amino acid residues near the ends (Arg^1^, Gly^2^, Ile^3^, Glu^13^, and Ile^14^) were preserved and no other candidates were considered because they were not in direct contact with the protein. No candidates were found in the first screening for Ala^9^ and Ala^10^, but Gly was also considered for these positions in the second screening. For other positions, one, two, or three residues with higher Ludi scores than the original ones were chosen. The chosen candidates are marked with * in Table 1. Note that all the original amino acid residues were included in the screening. In the second screening, the Ludi score was calculated for 3456 (=1 × 1 × 1 × 4 × 3 × 3 × 2 × 3 × 2 × 2 × 2 × 2 × 1 × 1) combinations of the candidate residues, whose top ten combinations are listed in Table 2. Note that the original sequence was only the 3362nd of the 3456 sequences investigated.

### 2.2. Enhancement of Antigen-Specific Antibody Production by the Peptide Obtained from In Silico Screening

**Figure 2:**
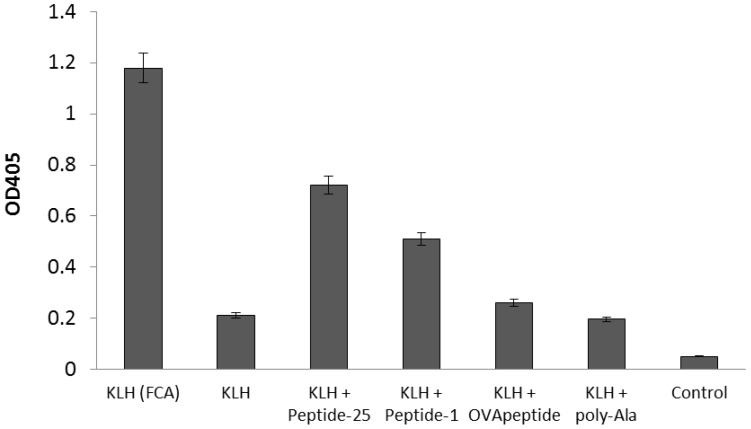
Potentiation of antigen-specific antibody production by agretope peptides. Serum titers (1000 × dilutions) from BALB/c mice immunized intraperitoneally with KLH, in the presence or absence of agretope or control peptides. Peptide-25 denotes a 15-mer peptide derived from Ag85B of *M. tuberculosis*. Peptide-1 denotes peptide RGIFFYVFAAYKEI, a 14-mer with the highest score from in silico screening. OVApeptide denotes peptide RGISQAVHAAHAEI, corresponding to amino acid residues 323–339 of ovalbumin. Poly-Ala denotes a control peptide composed of 15 alanines. Control denotes serum samples taken before immunization. Columns represent the average of three independent experiments, and error bars represent the standard deviation.

To determine the potentiation effect of the screened agretope peptide on antigen-specific antibody production, the keyhole limpet hemocyanin (KLH) antigen was injected intraperitoneally into BALB/c mice with or without the screened agretope peptide and Peptide-25. The peptide with the highest in silico binding affinity was synthesized and named Peptide-1. Serum titers against the antigen after immunization were measured (Figure 2). When mice were immunized with KLH in combination with FCA, a large increase of the titer against KLH was observed. When mice were immunized with KLH in Freund’s incomplete adjuvant (FIA), no increase in anti-KLH titers was observed. By contrast, co-immunization with Peptide-1 or Peptide-25 induced an increase in anti-KLH titers, with the latter having a larger effect. Co-immunization with the original OVApeptide induced only a small increase in the titer against KLH. These results show that the designed Peptide-1 could potentiate antigen-specific antibody production through in vivo immunization. Addition of control peptides (poly-Ala: a 15-mer of Ala) together with the antigen did not induce antigen-specific IgG antibodies.

### 2.3. Application of the Agretope Peptide to Establish Monoclonal Antibodies by In Vitro Immunization (IVI)

**Figure 3:**
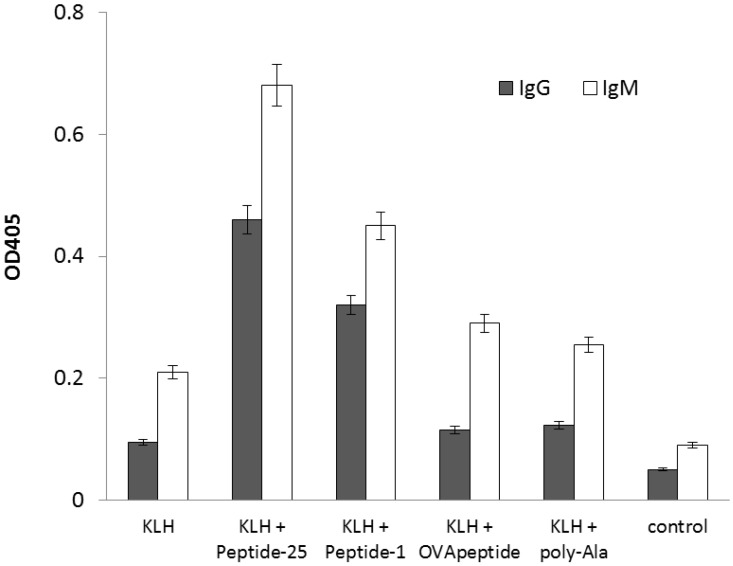
Induction of antigen-specific IgG by IVI in the presence or absence of agretope peptide. Both antigen-specific IgG and IgM were measured. Peptide-25 denotes a 15-mer peptide derived from Ag85B of *M. tuberculosis*. Peptide-1 denotes peptide RGIFFYVFAAYKEI, a 14-mer with the highest score from in silico screening. OVApeptide denotes peptide RGISQAVHAAHAEI, corresponding to amino acid residues 323–339 of ovalbumin. Poly-Ala denotes a control peptide composed of 15 alanines. Control denotes IVI without added antigen. Columns represent the average of three independent experiments, and error bars represent the standard deviation.

IVI offers a number of advantages over conventional immunization, such as using antigens otherwise toxic to host animals. However, the number of positive clones derived from IVI is limited, and the affinity of antibodies derived these clones is relatively low. Moreover, the majority of immunoglobulins produced in culture are IgMs instead of IgGs [15], which limits their application. Since FCA cannot be used in IVI, a proper immunopotentiator has yet to be found. Here, we used the agretope peptides to boost antibody production by IVI. After IVI with or without the agretope peptide, antigen-specific IgGs were measured from splenocytes’ supernatant. In the absence of the agretope peptide, very little antigen-specific IgG was produced (Figure 3). In contrast, Peptide-25 and Peptide-1 enhanced production of the antigen-specific IgG. These results confirm the capacity of the in silico designed agretope peptide to boost the induction of antigen-specific IgG in vitro.

**Figure 4:**
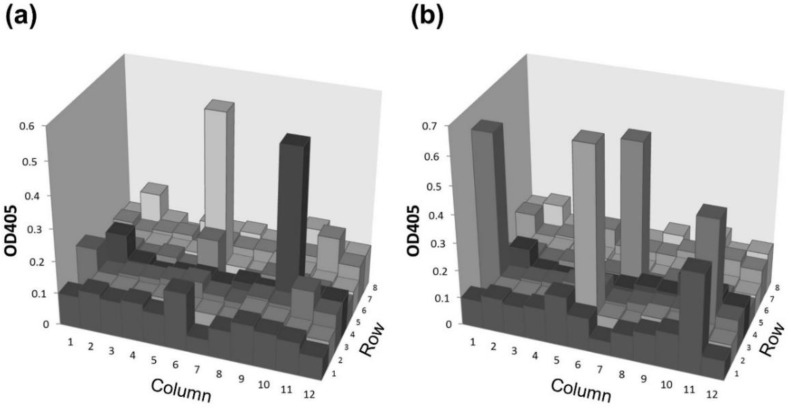
Levels of antigen-specific IgG from hybridomas generated by fusing myelomas with splenocytes from BALB/c mice immunized in vitro with Peptide-1. The supernatants from 96 randomly selected wells from the aforementioned 352 wells were collected, suspended into 96 well ELISA plate (12 columns × 8 rows) and analyzed for antigen binding activity of IgG (**a**) and IgM antibodies (**b**).

Finally, we attempted to establish a monoclonal antibody by IVI using the agretope peptide. After IVI, 5 × 10^7^ splenocytes were fused with 2.5 × 10^8^ myelomas for the formation of hybridomas. IVI and fusions were performed independently. The generated hybridomas were suspended in 96-well plates (*n* = 20) with limiting dilution to obtain one hybridoma colony per well, and the plates were incubated for seven days. Of the total 1920 wells (96 × 20), hybridoma colonies formed in 352 wells. The presence of antigen-specific IgG and IgM was measured in the supernatant from 96 randomly selected wells from the aforementioned 352 wells (Figure 4). Two colonies produced the antigen-specific IgG, whereas five colonies produced the antigen-specific IgM. The percentage of antigen-positive IgG and IgM clones was 2.1% and 5.2%, respectively. Without Peptide-1, the percentage of antigen-positive IgG and IgM clones was 0.93% and 3.56%, respectively [13]. Antigen-specific IgG antibody-producing cells increased over two-fold when Peptide-1 was added during IVI. The ratio of antigen-specific IgG-producing clones to antigen-specific IgM-producing clones was 0.4 with Peptide-1 and 0.26 without Peptide-1. Addition of Peptide-1 during IVI could induce the cells that produced the antigen-specific IgG.

## 3. Discussion

To induce antigen-specific antibody production, the antigen-specific B cell should be activated by the antigen-specific T cell. T cell activation is an indispensable step in antibody production. T cell receptors on the T cell surface cannot recognize extracellular antigens directly; instead, they recognize the agretope peptides (a part of antigen) that are presented by MHC-II molecules on macrophages and thus induce T cell activation. Then, activated T cells recognize agretope peptides presented by MHC-II molecules on the surface of B cells and activate these B cells. Activated B cells undergo cell divisions, enlargement, and differentiation to form a clone of antibody-secreting plasma cells. Therefore, intracellular processing of antigen to a peptide of suitable length and presentation of the digested antigen by the MHC-II are necessary for the activation of antigen-specific T cells. While extracellularly added antigens can be easily recognized by the antibody on the surface of B cells and induce the activation of the B cells, the same approach does not work for antigen-specific T cells. As a result, except when using attenuated viruses, antigen-dependent T cell activation cannot be easily induced experimentally. By designing peptides with high affinity for the MHC-II, suitable length, and appropriate sequence, it should be possible to entirely bypass the presentation process as these peptides would be expected to bind the empty MHC-II molecules and activate T cells. In the case of MHC-I molecules, agretope peptides are identified by in silico screening [16,17]. However, attempts to find agretope peptides with high affinity for MHC-II molecules have not been made yet. Here, we tried to find an agretope peptide that could bind tightly to the MHC-II and activate antibody production. Based on the crystal structure of a complex between MHC-II and agretope peptide, we performed in silico screening to identify the agretope peptide with the highest affinity for the MHC-II molecule.

The selected peptide was shown to boost antibody production both in vivo and in vitro. Not all retrieved sequences were examined due to the huge computational demand required for such a task and it is possible that potential candidates were overlooked. Nevertheless, the results obtained with Peptide-1 suggest that the present strategy of in silico screening was fairly successful and we could identify some strong binders from the library.

From our in silico screening, the 12th amino acid (R12) of all top ten peptides was Lys, which has a positive charge and relatively large size. The 4th, 5th, and 8th amino acids (R4, R5, and R8) were Phe. The side chains of amino acids at these positions, especially R5 and R8, are facing the outside of the peptide binding pocket on MHC-II molecules. Accordingly, Phe might be selected simply for its space filling effect. The 4th, 7th, 9th, and 12th amino acids (R4, R7, R9, and R12) in the agretope peptide are thought to be the anchor residues responsible for binding to the MHC-II pocket. These amino acids share some common features among experimentally determined agretope peptides [18]. For example, R4 and R7 correspond often to Tyr, Ile, Trp, Val, or Phe; R9 to Gln, Asn, or Ala; and R12 to Lys or Arg. Based on the screening, Phe was selected for position R4, Val for R7, Ala for R9, and Lys for R12. These in silico results agree with the experimental observations.

In the second screening, the Ludi score was calculated for 3456 combinations of the candidate residues. Surprisingly, the original sequence was only the 3362nd of the 3456 sequences investigated. Peptides corresponding to original sequences had only limited effect on the experimental potentiation of antibody production. Original amino acid residues near the ends (Arg^1^, Gly^2^, Glu^13^, and Ile^14^) were unchanged during in silico simulation because they were not in direct contact with the pocket in MHC-II. It could, nevertheless, be possible to change these amino acids without affecting immune system activation. For an efficient activation of the immune system it is also important that the synthetic peptide remains stable in solution even at a high concentration. For a stronger activation, it is worth trying to change the terminal amino acid to an amino acid with high polarity but without charge, such as Ser, Thr, Asn, and Gln.

The peptide derived from in silico screening was applied to establish monoclonal antibodies by IVI. IVI is suitable for the generation of many types of antigens, including those that are problematic for in vivo immunization. Moreover, as it does not involve injection of animals with the antigen, IVI avoids the risk of antigen toxicity. With IVI, immune cells isolated from naïve animals are stimulated with an antigen in vitro, thereby resulting in the induction of B cells that produce antigen-specific antibodies [15]. Subsequently, such cells are fused with myeloma cells to form hybridomas that produce the desired antigen-specific monoclonal antibodies. With further development, IVI should become especially useful for obtaining human monoclonal antibodies [19]. If one can establish human monoclonal antibodies with IVI using human peripheral blood mononuclear cells [20], complicated processes, such as humanization or chimerization of antibodies [21], could be avoided. Nevertheless, IVI has not been used widely until now, because its procedures are complex and results have been unsatisfactory. In particular, the majority of IVI-derived clones produce IgMs, which show suboptimal affinity for broader usage [15]. Consequently, useful antibodies with high affinity are rarely obtained with conventional IVI protocols. To date, potent immunostimulants such as CpG ODN, MDP, interleukin (IL)-2, and IL-4, have been applied successfully with IVI. However, most protocols still fail to deliver sufficient stimuli to antigen-specific B cells for their expansion and/or differentiation into antibody-producing cells.

We previously developed an IVI protocol that effectively activated immune cells via two-step antigen stimulation [22]. This protocol enables the induction of antigen-specific IgG production. Culture conditions, including cell density, type of stimulant, and initial cell preparation, were found to be important for inducing the IgG response. In addition, an analysis of the genes and cytokines expressed during IVI, showed that antigen-specific B cells were activated specifically via CD4-positive helper T cells. However, the number of positive clones from this improved IVI is still not sufficient to obtain monoclonal IgG antibodies with the desired characteristics. Here, we applied this protocol to assess antibody production in response to the identified synthetic peptides. This protocol consists of two cycles of repeated antigen stimulation followed by cell expansion, which increases the frequency of plasma cells that produce antigen-specific IgG antibodies. The effect of the agretope peptide was most remarkable when the peptides were applied during antigen stimulation in the first cycle. As evidence for this concept, our IVI protocol with Peptide-1 enabled us to establish an IgG antibody against KLH. This shows that a peptide screened in silico could be used as immunostimulant in IVI, which would induce class-switching [15,23]. However, future studies will need to confirm the increase in affinity due to affinity maturation [24,25] induced by agretope peptides. If these two processes, both of which are essential for obtaining antibody-producing cells in vivo, are achieved with IVI, IVI could become a powerful tool for creating high-affinity monoclonal antibodies. Critically, a screened and selected peptide is advantageous over other immunostimulants, as it could be applied without evoking the harsh inflammation damage to the host animal caused by in vivo immunization.

We showed previously that Peptide-25 derived from Ag85B of *M. tuberculosis* activated T cells by modulating the Th1/Th2 balance during immunization and thus increased the number of antigen-specific antibody-producing cells [13]. This potentiation was remarkably high in BALB/c mice. For the peptide derived in this study, it will be interesting to determine the effect on the different MHC-II classes using various mouse strains. Seemingly, the mechanism of potentiating antibody production by the peptide derived in this study awaits further investigation. The difference in activation mechanism between Peptide-25 and agretope peptides obtained in this study would suggest important factors for designing more efficient peptides.

## 4. Materials and Methods

### 4.1. Mice and Immunization

Approximately 6–8-week-old female BALB/c mice were obtained from SLC (Tokyo, Japan). The mice received intraperitoneal injections of either 10 μg KLH (Thermo Fisher Scientific, Waltham, MA, USA) in FCA (Sigma-Aldrich, St. Louis, MO, USA) or 10 μg KLH with or without 10 μg of peptides in FIA (Sigma-Aldrich) in a volume of 0.2 mL. Thirty days after immunization, 200 μL of blood was collected from the tail; serum was prepared from the blood, and its titer against the antigen was measured using enzyme-linked immunosorbent assay (ELISA). All animals were cared for and maintained in accordance with the guidelines of the National Institute of Advanced Industrial Science and Technology (AIST). Project was approved by the committee for the Experiments involving Animals of AIST (Project identification code: A2012-033, April 2012).

### 4.2. In Silico Screening

In silico screening was performed with Cerius 2 software (Accelris Inc., San Diego, CA, USA) using the AutoLudi method, an automated algorithm that designs a ligand for a protein whose 3D structure is known [26,27,28]. In the third version of AutoLudi (Ludi-3, [28]), the change in free energy (ΔG) upon peptide ligand binding is calculated as follows:

ΔG_binding_ = ΔG_0_ + ΔG_polar_ + ΔG_apolar_ + ΔG_solv_ + ΔG_flexi_,

where ΔG_0_ is a contribution to the binding energy independent of any specific interactions with the protein, possibly a reduction of binding energy due to overall loss of translational and rotational entropy of the ligand, ΔG_polar_ represents polar interactions such as H-bonds and ionic bonds, ΔG_apolar_ denotes apolar interactions comprising lipophilic interactions and interactions between aromatic rings, ΔG_solv_ corresponds to the desolvation effect, and ΔG_flexi_ describes the loss of binding energy due to fixation of the rotational freedom of the ligand.

△G is then converted to the Ludi score:

Score = −73.33△G (*T* = 298 K)

As the Ludi score is directly related to the dissociation constant (*Ki*) according to the formula Score = 100 log*Ki*, the score can be used as the criterion for ligand binding affinity.

### 4.3. Synthetic Peptides

Synthetic peptides were prepared by Sigma-Aldrich Japan (Tokyo, Japan). These peptides were dissolved in distilled water or 100 mM Tris–HCl (pH = 7.4) to a final concentration of 1 mg/mL.

### 4.4. IVI

Six-week-old female BALB/c mice were obtained from SLC. The mice were sacrificed and their spleens were removed aseptically [22]. The spleens were squeezed and single-cell suspensions were prepared. The cells were washed once in RPMI-1640 (Sigma Aldrich), and re-suspended in 10 mL RPMI-1640 medium containing 10% fetal bovine serum (FBS). Erythrocytes and granulocytes were removed using Lympholyte-M (Cedarlane Laboratories, Burlington, ON, Canada). CD8-positive T cells and natural killer cells were removed from splenocytes by negative selection methods using anti-CD8 and anti-CD49b antibody-coated magnetic beads (Miltenyi Biotech, San Diego, CA, USA), according to the manufacturer’s instructions. For IVI, the cells were washed twice and incubated individually at 37 °C for two days in RPMI-1640 containing 20% FBS, with 1 μg KLH as antigen, and with or without 3 μg agretope peptides plus 0.25 μM CpG ODN (5′-tccatgacgttcctgacgtt-3′; Hokkaido System Science Co. Ltd., Sapporo, Japan) or 0.25 μM MDP (Sigma Aldrich) as stimulants. The stimulated cells were collected by centrifugation and then expanded in culture medium with IL-2 at 10 ng/mL, IL-4 at 2.5 ng/mL, and IL-21 at 10 ng/mL (PeproTech Inc., Rocky Hill, NJ, USA) for two days. For secondary antigen stimulation, these expanded cells were collected by centrifugation and stimulated with 0.25 μM KLH, 0.08 μM CpG ODN, and 0.25 μM MDP. Antigen-specific IgG antibody levels in culture supernatants of stimulated splenocytes were determined by ELISA.

### 4.5. ELISA

A 96-well ELISA plate was coated with 50 μL of 5 μg/mL KLH per well. A blocking solution (Blocking Reagent for ELISA; Roche, Basel, Switzerland) was applied, and the plate was incubated for 2 h [29,30]. The plate was subsequently washed with PBS containing 0.05% Tween-20 (PBS-T). Afterwards, 50 μL of 1000 × diluted serum from immunized mice or supernatant from in vitro-stimulated splenocytes was added to each well. After the wells were washed, alkaline-phosphatase-labeled anti-mouse IgG antibody or alkaline-phosphatase-labeled anti-mouse IgM antibody (Chemicon International, Temecula, CA, USA) was added. The amount of antigen-specific antibody was measured using an alkaline-phosphatase substrate kit (Sigma Aldrich), and the plates were read at 405 nm using a Model 680 microplate reader (Bio-Rad Laboratories, Hercules, CA, USA). All experiments were conducted twice, and the average signal intensity was used in the analysis.

### 4.6. Hybridoma Formation and Quantification of Positive Clones by IVI

To establish monoclonal antibodies against KLH by IVI, splenocytes from BALB/c mice immunized with KLH in vitro were isolated and mixed with an equal number of P3 × 63Ag8U.1 (P3-U1) murine myeloma cells. Cells were routinely cultured in RPMI-1640 supplemented with 10% FBS at concentrations between 1 × 10^5^ and 1 × 10^6^ cells/mL. The two cell types were counted using a hemocytometer and mixed together at a 1:1 ratio. The cells were fused by PEG methods. Next, the cell suspensions were pipetted into 10 mL RPMI-1640 medium (free of phenol red) containing 20% FBS and 5% Briclone (QED Bioscience Inc, San Diego, CA, USA). The fused cells were suspended in a 96-well plate and grown at 37 °C under a 5% CO_2_-enriched atmosphere. After 24 h, hypoxanthine (Sigma-Aldrich) medium was added to each well. The generated hybridomas were incubated in a 96-well plate for seven days. Supernatants from the individual wells were screened by ELISA.

## 5. Conclusions

In silico screening was performed to find a 14-mer peptide with high affinity for MHC-II. Peptide sequences with higher affinity were identified. The peptide expected to have the highest affinity was synthesized and its effect on antibody production was verified experimentally. Co-immunization of peptide with antigen increased antigen-specific IgG antibody production, though the potentiation effect was smaller than that of Peptide-25. Using this peptide as immunostimulant, monoclonal IgG antibodies could be established by in vitro immunization.

## Figures and Tables

**Table 1:** Result of the first AutoLudi screening. For each amino acid position of the 14-mer peptide, amino acid residues with higher Ludi score than Gly are listed in the descending order of the score along with the original residue. Those residues with Ludi scores higher than the original ones are shown in bold face. The amino acid residues chosen manually for the subsequent second screening are marked with an asterisk (*).

Residue Position	Original	Rank 1	Rank 2	Rank 3	Rank 4	Rank 5	Rank 6	Rank 7
1	Arg *	Lys	Trp	Tyr	Met			
2	Gly *	**Tyr**	**Phe**	**Met**	Ser	Thr		
3	Ile *	**Lys**	**Tyr**	**Phe**	**Thr**	**Ser**	**Ile**	**Met**
4	Ser *	Phe *	Leu *	Ile *	**Val**			
5	Gln *	Phe *	Lys *	**Met**				
6	Ala *	Tyr *	Gln *	**Met**				
7	Val *	Thr *	Ser					
8	His *	Phe *	Lys *	**Glu**	**Trp**	**Met**	**Ile**	**Val**
9	Ala *	(Gly *)						
10	Ala *	(Gly *)						
11	His *	Tyr *	**Phe**	Met	Ile	Leu	Val	Lys
12	Ala *	Lys *						
13	Glu *	**Met**	**Ile**	**Leu**	**Val**			
14	Ile *	Met						

**Table 2:** Result of the second AutoLudi screening. The top ten peptide sequences with the highest Ludi score are listed. * The original sequence.

Rank	Score	R1	R2	R3	R4	R5	R6	R7	R8	R9	R10	R11	R12	R13	R14
1	1649	Arg	Gly	Ile	Phe	Phe	Tyr	Val	Phe	Ala	Ala	Tyr	Lys	Glu	Ile
2	1620	Arg	Gly	Ile	Phe	Phe	Tyr	Val	Phe	Gly	Ala	Tyr	Lys	Glu	Ile
3	1620	Arg	Gly	Ile	Phe	Phe	Gln	Val	Phe	Ala	Ala	Tyr	Lys	Glu	Ile
4	1617	Arg	Gly	Ile	Phe	Phe	Tyr	Thr	Phe	Ala	Ala	Tyr	Lys	Glu	Ile
5	1613	Arg	Gly	Ile	Phe	Phe	Tyr	Val	Phe	Ala	Gly	Tyr	Lys	Glu	Ile
6	1610	Arg	Gly	Ile	Phe	Phe	Tyr	Val	Phe	Ala	Ala	His	Lys	Glu	Ile
7	1605	Arg	Gly	Ile	Phe	Phe	Tyr	Val	His	Ala	Ala	Tyr	Lys	Glu	Ile
8	1597	Arg	Gly	Ile	Phe	Phe	Tyr	Val	Lys	Ala	Ala	Tyr	Lys	Glu	Ile
9	1593	Arg	Gly	Ile	Phe	Phe	Gln	Val	Phe	Gly	Ala	Tyr	Lys	Glu	Ile
10	1588	Arg	Gly	Ile	Phe	Phe	Tyr	Thr	Phe	Gly	Ala	Tyr	Lys	Glu	Ile
-															
3362 *	968	Arg	Gly	Ile	Ser	Gln	Ala	Val	His	Ala	Ala	His	Ala	Glu	Ile
